# Differences in the Comparative Stability of Ebola Virus Makona-C05 and Yambuku-Mayinga in Blood

**DOI:** 10.1371/journal.pone.0148476

**Published:** 2016-02-05

**Authors:** Michael Schuit, David M. Miller, Mary S. Reddick-Elick, Carly B. Wlazlowski, Claire Marie Filone, Artemas Herzog, Leremy A. Colf, Victoria Wahl-Jensen, Michael Hevey, James W. Noah

**Affiliations:** 1 National Biodefense Analysis and Countermeasures Center, Frederick, MD, United States of America; 2 Censeo Consulting Group, Washington, DC, United States of America; 3 Department of Homeland Security (DHS) Science and Technology Directorate, Washington, DC, United States of America; Division of Clinical Research, UNITED STATES

## Abstract

In support of the response to the 2013–2016 Ebola virus disease (EVD) outbreak in Western Africa, we investigated the persistence of Ebola virus/H.sapiens-tc/GIN/2014/Makona-C05 (EBOV/Mak-C05) on non-porous surfaces that are representative of hospitals, airplanes, and personal protective equipment. We performed persistence studies in three clinically-relevant human fluid matrices (blood, simulated vomit, and feces), and at environments representative of in-flight airline passenger cabins, environmentally-controlled hospital rooms, and open-air Ebola treatment centers in Western Africa. We also compared the surface stability of EBOV/Mak-C05 to that of the prototype Ebola virus/H.sapiens-tc/COD/1976/Yambuku-Mayinga (EBOV/Yam-May), in a subset of these conditions. We show that on inert, non-porous surfaces, EBOV decay rates are matrix- and environment-dependent. Among the clinically-relevant matrices tested, EBOV persisted longest in dried human blood, had limited viability in dried simulated vomit, and did not persist in feces. EBOV/Mak-C05 and EBOV/Yam-May decay rates in dried matrices were not significantly different. However, during the drying process in human blood, EBOV/Yam-May showed significantly greater loss in viability than EBOV/Mak-C05 under environmental conditions relevant to the outbreak region, and to a lesser extent in conditions relevant to an environmentally-controlled hospital room. This factor may contribute to increased communicability of EBOV/Mak-C05 when surfaces contaminated with dried human blood are the vector and may partially explain the magnitude of the most recent outbreak, compared to prior outbreaks. These EBOV persistence data will improve public health efforts by informing risk assessments, structure remediation decisions, and response procedures for future EVD outbreaks.

## Introduction

In March 2014, the World Health Organization was notified of an outbreak of a communicable disease characterized by fever, severe diarrhea, vomiting, and a high fatality rate in Guinea. The etiological agent was subsequently identified as a novel Ebola virus (EBOV) variant [1]. This Ebola virus disease (EVD) outbreak was unprecedented in geographic location, spread to multiple countries, number of infections/deaths, and duration. As of January 10, 2016, the Centers for Disease Control and Prevention (CDC) reported 28,637 EVD cases and 11,315 deaths, with widespread transmission of EVD in three countries: Guinea, Liberia, and Sierra Leone [2]. There were also multiple cases diagnosed in or evacuated to the U.S., including two U.S.-acquired cases in healthcare workers [3]. Although there has been a decrease in the number of new case reports, a low level of sustained EVD transmission was still occurring during the week of Jan 15, 2016 [4], as demonstrated by a new case report in Sierra Leone.

Genetic sequencing revealed the Guinean Ebola virus variant responsible for the recent outbreak (Ebola virus/H.sapiens-tc/GIN/2014/Makona) has 97% identity to EBOV variants from the Democratic Republic of Congo and Gabon [5]. Although it is related to the prototype Ebola virus/H.sapiens-tc/COD/1976/Yambuku-Mayinga (EBOV/Yam-May) and within the *Zaire ebolavirus* species, phylogenetic analyses of the full-length sequences places EBOV/Makona in a separate, basal position within the EBOV clade, due to almost 400 substitutions or single-nucleotide polymorphisms [5]. The unprecedented size of the recent EVD outbreak has raised questions as to why this outbreak was different from previous, much smaller outbreaks. Divergent societal/cultural practices in a region not previously affected by EVD have likely contributed to the greater magnitude of this outbreak [6]. However, contributions due to differences in the virus sequence and resulting phenotype (such as increased environmental stability and/or resistance to inactivation methods compared to EBOV from previous outbreaks) cannot be ruled out, and can be directly investigated via laboratory studies [7].

Because of the large size of the recent outbreak and the fact that transmission of EBOV has historically been through direct contact with infectious fluids [8], the empirical testing of the phenotypic fitness of the recent outbreak EBOV variant, specifically its persistence in the blood, secretions, organs or other bodily fluids, and in environments contaminated with such fluids, is of substantial interest [9], as is how long contaminated surfaces can potentially serve as a source of transmission of the virus. This information is important for the management of future EBOV outbreaks, as well as for informed risk assessments for the protection of health care workers, first responders, and the general population. To address this data gap, we determined the duration of EBOV infectivity in three dried human body fluid matrices on inert, non-porous surfaces commonly associated with hospitals, airplanes, and personal protective equipment (PPE) surfaces, and evaluated how environmental factors influence EBOV survival. Our data show that EBOV persistence is environment- and matrix-dependent, and that, in clinically-relevant matrices, EBOV persists longest in dried human blood under environmental conditions common in the outbreak region. Lastly, we show that the recent outbreak virus variant EBOV/Mak-C05 is more stable during drying in blood than a previous outbreak virus variant (EBOV/Yam-May), which may contribute to extended persistence of the 2013–2016 outbreak virus variant in blood, compared to previous outbreak variants.

## Materials and Methods

### Cells and cell culture

Vero E6 (*Chlorocebus aethiops* kidney; ATCC (Manassas, VA, # CRL-1586)) cells were used for *in vitro* propagation of EBOV and microtitration assays. Cells were grown in complete growth media (cell culture media) consisting of Minimum Essential Medium (Life Technologies, Frederick, MD, # 11095–098) supplemented with heat-inactivated 10% fetal bovine serum (Sigma Aldrich, St. Louis, MO, # 12107C), 2 mM Glutamax (Life Technologies # 35050–061), 0.1 mM NEAA (Life Technologies # 11140–050), 1 mM sodium pyruvate (Life Technologies # 11360–070) and 1% antibiotic-antimycotic solution (Life Technologies # 15240–062). Cells were maintained as adherent cell lines at 37°C in a humidified 5% CO_2_ atmosphere and were passaged as needed and harvested from flasks using TrypLE Express Enzyme (Life Technologies # 12604–013).

### Virus stock preparation and characterization

Ebola virus/H.sapiens-tc/GIN/2014/Makona-C05 (EBOV/Mak-C05) was a kind gift from Dr. Heinz Feldmann, Rocky Mountain Laboratory/NIAID/NIH. Passage one material was provided as cell culture supernatant and used to generate the virus stock that was used for this study. Ebola virus/H.sapiens-tc/COD/1976/Yambuku-Mayinga (EBOV/Yam-May) was obtained from the Centers for Disease Control and Prevention and virus stocks were prepared in the same manner. All work with viable EBOV was performed in the NBACC BSL-4 laboratory. Virus stocks were prepared in Vero E6 cells, and confirmed negative for mycoplasma and endotoxin. Mycoplasma testing was performed using a MycoAlert PLUS Mycoplasma Detection Kit (Lonza, Walkersville, MD, # LT07-701,) according to manufacturer’s instructions. Endotoxin testing was performed using a Pierce LAL Chromogenic Endotoxin Quantitation Kit (Life Technologies, Grand Island, NY, # 88282) according to manufacturer’s instructions.

### Test surfaces

Test surfaces were obtained from the sources indicated below and cut into 22 mm^2^ coupons: Stainless steel (grade 304, Diamond Perforated Metals, Visalia, CA); TyChem QC (Dupont, Wilmington, DE); nitrile (Kimberly-Clark Sterling Nitrile Exam Gloves # 5070, ThermoFisher Scientific, Rockville, MD # 19-048-133); Polypropylene (Piedmont Plastics, Richmond, VA). All surface coupons were sterilized by irradiation with gamma rays (2.5 Mrad) before use in the study.

### Test matrices

Whole human blood, CD14-depleted human blood [10], and feces were obtained from Bioreclamation IVT (Westbury, NY). Feces was obtained as a pool of loose stool from 25 different donors. Feces samples were sterilized by irradiation with gamma rays (4 Mrad), centrifuged briefly (500 x g for 5 min) to remove particulates, and the resulting supernatant used in the study. Simulated gastric fluid (SGF) consisting of 0.2% (w/v) sodium chloride in 0.7% (v/v) hydrochloric acid was obtained from ThermoFisher Scientific. Pepsin (3.2 g/L, Sigma Aldrich # P6887) was added on the day of use and the SGF with pepsin was filtered. To simulate the conditions of the human GI tract following ingestion of food, the stability of EBOV was examined utilizing SGF that has been buffered by the addition of a volume of milk. A gastric volume (GV) to milk ratio of 1:1.36 was used based on an average GV in fasting adults of 173 mL [11] and a volume of 236 mL (one cup) for a single serving of milk. [12]. The final pH of the simulated gastric fluid buffered with milk was not measured. For the study, cell culture-amplified virus stock (10^7.4^ TCID_50_/mL) was spiked 1:10 into each test matrix immediately prior to deposition onto test surface coupons.

### Virus microtitration assay

Virus controls and virus recovered from test surface coupons were serially-diluted (10^0^–10^5^) in 96-well microtiter plates containing confluent Vero E6 cell monolayers. Dilutions were carried out using the Precision Microplate Sample Processor and Pipetting system (BioTek Instruments, Inc., Winooski, VT, # PRC384U) setup in 8-channel pipette mode. Seven replicates of each dilution were performed. Primary containment was maintained throughout the assay with virus being loaded in the first well inside a standard Class IIA2 biosafety cabinet and then transferred to a larger Class IIA2 BioPROTECT containment enclosure (Baker Company, Sanford, ME, Model #VR2) where two Precision systems performed the dilutions. The plates were incubated at 37°C in a passively humidified 5% CO_2_ atmosphere for up to 14 days. The plates were visually observed for the presence of virus-induced cytopathic effects (CPE) at each dilution compared to a negative (media only) control or a matrix (media + matrix only) control. The viral dilution leading to CPE in 50% of inoculated wells was visually estimated using the Reed-Muench method [13], with an error of ±0.5 Log TCID_50_/mL and a limit of detection of 0.7 Log TCID_50_/mL.

### Quantitative reverse-transcription real-time PCR

The total number of recovered virus genomic copies was determined using a quantitative reverse transcriptase real-time PCR assay. RNA from test samples for qPCR analysis was isolated by TRIzol extraction using manufacturer’s instructions, followed by purification by chromatography [14]. PCR reagents were obtained from the Critical Reagents Program [15]. Briefly, 5 μL of purified sample RNA was combined with 15 μL of reagent cocktail (containing proprietary primers, FAM-probe, and reaction reagents) and run in an ABI-7500 PCR machine using the following amplification protocol: reverse transcription (1 cycle, 15 min/50°C); Taq inhibitor inactivation (1 cycle, 2 min/95°C); PCR amplification (45 cycles, 15 sec/95°C, 60 sec/55°C). A standard curve using a known copy number of EBOV NP RNA was run with each reaction set.

### Data statistical analysis

The primary response variable of this study was Log TCID_50_/mL. Because this variable has an implied logarithmic transformation, the decay function can be written as:

C=β1t+β0

where *C* is the viable concentration at time *t* (given in Log TCID_50_/mL), *β*_*1*_ is the decay rate, and *β*_*0*_ is the initial viable concentration at time 0. The decay rates were estimated using ordinary least squares for each treatment considered during the experiment. The 95% confidence intervals for the decay rates were also calculated. Matlab software (MathWorks, Natick, MA) was used for statistical analyses.

Analysis of Variance (ANOVA) was used to determine which experimental factors (i.e. surface, matrix, and environment) had a significant effect on the decay rate. Results are reported at a 5% significance level. Data acquired from simulated vomit and feces were removed prior to ANOVA analysis because the fast viability loss in these two matrices made it difficult to estimate reliable decay rates. ANOVA was also used to determine which experimental factors had a significant effect on the viability loss due to drying. A similar analysis was also performed on the intercept terms (β_0_) to show that there was no significant difference in initial viable concentrations across the test surfaces. Because no test surface dependence was evident, data were pooled across the test surfaces and a composite decay rate was calculated for each matrix/environment/variant combination and 95% confidence intervals were calculated for the decay rate using the pooled data. Viability decay rates were derived from the slope of the line fitted to data points from day 0 (post-dry) to the limit of microtitration assay virus detection (0.7 Log TCID_50_/mL). Data points where the mean virus Log TCID_50_/mL viability value first intersected the limit of virus detection were included and valued at 0.7 Log TCID_50_/mL, and subsequent points were not used for decay rate calculation.

### Environmental survey of representative environmental temperatures and humidity

Environmental conditions that are representative of in-flight airline cabins were derived from published literature [16, 17]. Environmental conditions that are representative of environmentally-controlled hospital interiors were taken from ASHRAE (American Society of Heating, Refrigerating, and Air-Conditioning Engineers) Standard Indoor Conditions [18]. Environmental conditions that are representative of open air facilities in the outbreak region of Western Africa were derived from a survey of NOAA (National Oceanic and Atmospheric Administration) weather data from weather stations in Conakry, Guinea; Freetown, Sierra Leone; and Monrovia, Liberia over the last five years, from which the most frequently occurring temperature and relative humidity combinations were identified. Although three different environmental conditions were targeted (21°C/15% RH representing an in-flight airline cabin [16, 17], 21°C/40% RH representing an interior, environmentally-controlled hospital room [18], and 28°C/85% RH representing an open-air EBOV treatment facility in Western Africa, actual experimental test condition means and standard deviations were slightly different to due equipment limitations (22±1°C/17±5.0% RH, 22±1°C/41±1.0% RH, and 28±1°C/90±3% RH).

### Study design and experimental protocol

**Fig 1:**
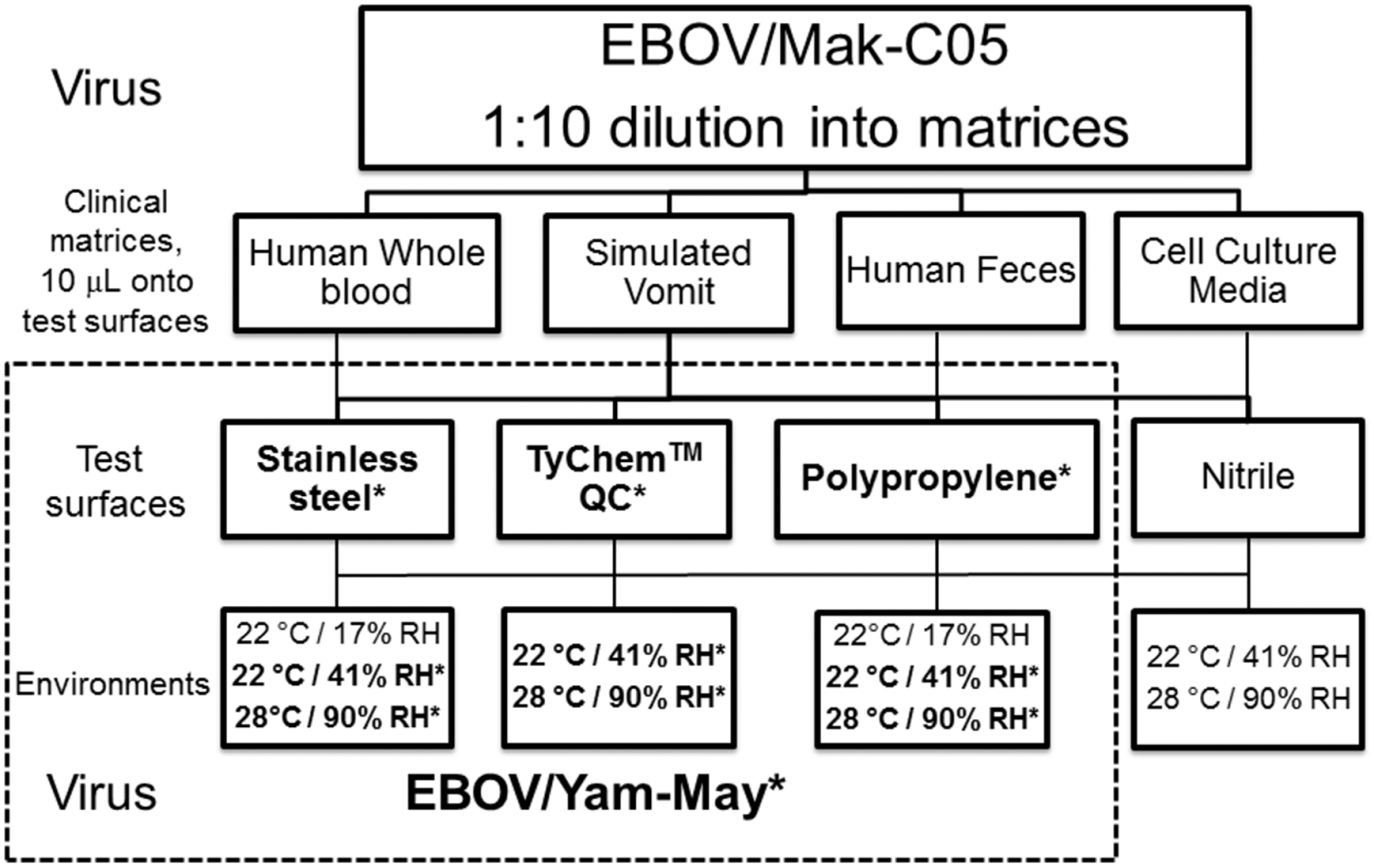
EBOV Surface Persistence Study Design. Vero E6-cultured stocks of EBOV/Mak-C05 were diluted 1:10 into the clinical test matrices. Ten μL drops (containing 10^6.4^ TCID_50_/mL) of each virus/matrix mixture were deposited onto sterile test surface coupons, dried 1–4 hours (environment dependent) in an environmentally-controlled chamber, and then aged in the same chamber at the indicated environment for 3–10 days. Approximately 10^4.4^ (~25,000) TCID_50_ were deposited on each test surface coupon. Viable virus was recovered from surface coupons immediately after deposition (wet), immediately after drying, and at a minimum of three subsequent timepoints. At each timepoint, triplicate samples were analyzed for virus viability by microtitration assay. Four hospital and PPE-associated surfaces were tested against multiple environments. For comparison, a subset of surfaces and environmental conditions (indicated by dotted box, bolded font, and an *) were used to determine the comparative persistence of EBOV/Yam-May.

We designed the study to measure the surface persistence of Ebola virus/H.sapiens-tc/GIN/2014/Makona-C05 (EBOV/Mak-C05) in three clinically-relevant human fluid matrices (whole human blood, human feces, and simulated vomit). We also examined the viability decay rate of EBOV/Mak-C05 in cell culture media to allow for data comparison with previously published studies [19–21]. Fig 1 shows the EBOV surface persistence study design. We examined virus persistence on four non-porous test surfaces (stainless steel, TyChem QC, polypropylene plastic, and nitrile) by depositing drops (10 μL) of virus-spiked matrix onto individual test surface coupons, which were dried and aged at the indicated environment. The concentration of virus in the matrices (10^6.4^ TCID_50_/mL) was derived by diluting virus stock (at 10^7.4^ TCID_50_/mL) 1:10 into each test matrix. This virus concentration represents the upper limit of infectious viremia that has been reported in blood samples evaluated from the 1976 outbreak [22] and is a relevant concentration in blood. The study with EBOV/Mak-C05 evaluated all matrices and all surfaces at 22°C/41% RH and 28°C/90% RH, which represent interior hospital and exterior Western Africa environments, respectively. We also examined virus persistence in all matrices at 22°C/17% RH (which represents the interior environment of an in-flight airline cabin), but only on stainless steel and polypropylene surfaces (as these represent relevant surfaces found in airplanes). For comparison to EBOV/Mak-C05, we measured the surface persistence of Ebola virus/H.sapiens-tc/COD/1976/Yambuku-Mayinga (EBOV/Yam-May) in all matrices and on a subset of surfaces (stainless steel, TyChem QC, and polypropylene plastic) at 22°C/41% RH and 28°C/90% RH. To reduce the size of the comparative study while still generating a statistically-supported data set, EBOV/Yam-May persistence was not examined on nitrile or at the airline cabin environment. For all studies, virus in matrix was recovered from surface coupons prior to drying, immediately post-drying, and at various timepoints from 12 to 240 h post-drying, after which viability persistence (decay rates) was determined. Lastly, we used qRT-PCR to determine total virus input and recovered genomic copies from a subset of the wet and dried EBOV/Mak-C05 samples, which verified efficient and quantitative virus recovery from the test surface coupons. The raw data for all experiments can be found in Tables A-G in S1 File.

To perform these experiments, test surface coupons were aseptically placed in six-well microplates. A single drop containing 10 μL of each matrix spiked with virus (final concentration of 10^6.4^ TCID_50_/mL) was applied to individual surface coupons and the coupons were placed in the targeted test environmental ranges (21°C/15% RH, 21°C/40% RH, 28°C/85% RH) in an environmental chamber (KB09, Darwin Chamber Company, St. Louis, MO) for 1–4 four hours to dry. Actual test conditions were 22.0±0.1°C/17.0±5.0% RH, 22.0±1.0°C/41.0±1.0% RH, and 28.0±0.1°C/90.0±0.6% RH. At the indicated time-points, (pre-dry and immediately post-drying on days 0, and days 1–10 post-drying), the coupons were submerged in 5 mL of cell culture media and then vortexed for 30 seconds to recover the matrix/virus application. The amount of viable virus in the recovery media (recovered Log TCID_50_/mL) was determined using the microtitration assay. Each test surface timepoint was performed in triplicate. Four studies were performed. One study (Table D in S1 File) was performed at 22°C/17% RH for EBOV/Mak-C05, using stainless steel and polypropylene surfaces and matrices, for 72 h. One study (Tables A and E in S1 File) was performed at 22°C/41% RH for EBOV/Mak-C05 and EBOV/Yam-May, using all surfaces for EBOV/Mak-C05 and stainless steel, TyChem QC, and polypropylene surfaces for EBOV/Yam-May, and all matrices, for 72–96 h. One study (Tables B and F in S1 File) was performed at 28°C/90% RH for EBOV/Mak-C05 and EBOV/Yam-May, using all surfaces for EBOV/Mak-C05 and stainless steel, TyChem QC, and polypropylene surfaces for EBOV/Yam-May, and all matrices, for 72 h. Finally, one verification study (Tables C and G in S1 File) was performed at 28°C/90% RH for EBOV/Mak-C05 and EBOV/Yam-May, using stainless steel, TyChem QC, and polypropylene surfaces, and cell culture and blood matrices, for 240 h.

## Results

### EBOV/Mak-C05 persists longest in dried whole blood at higher relative humidity

**Fig 2:**
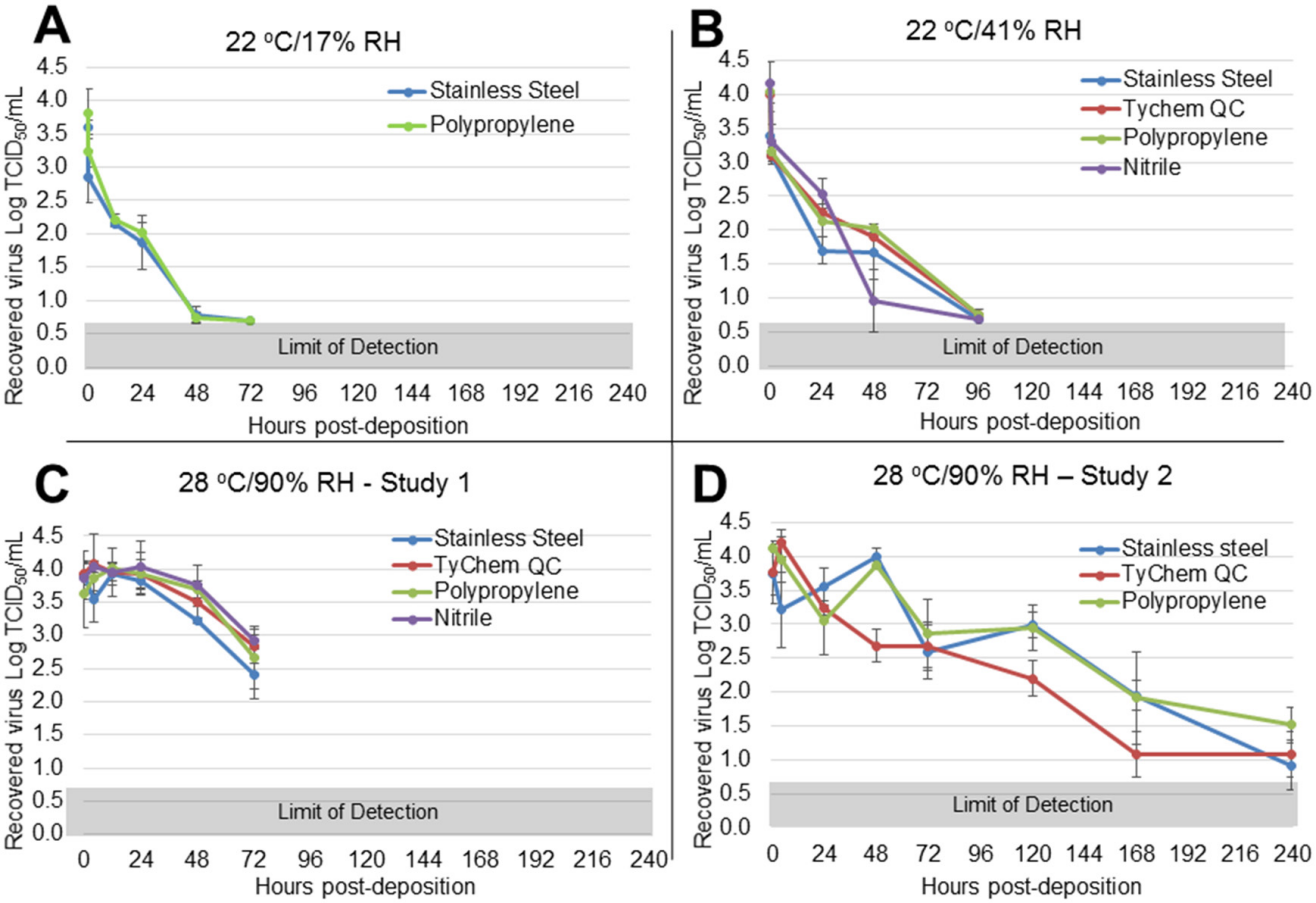
EBOV/Mak-C05 Surface Persistence in Blood at Three Different Environments. The surface-specific decrease in EBOV/Mak-C05 viability in human whole blood was measured at (A) 22°C/17% RH (stainless steel and polypropylene only), (B) 22°C/41% RH, or (C-D) 28°C/90% RH. The microtitration assay limit of detection (0.7 Log TCID_50_/mL) is indicated by a gray bar at the bottom of each graph. The graphs in panels C-D are derived from two separate studies, as described in the methods section.

**Fig 3:**
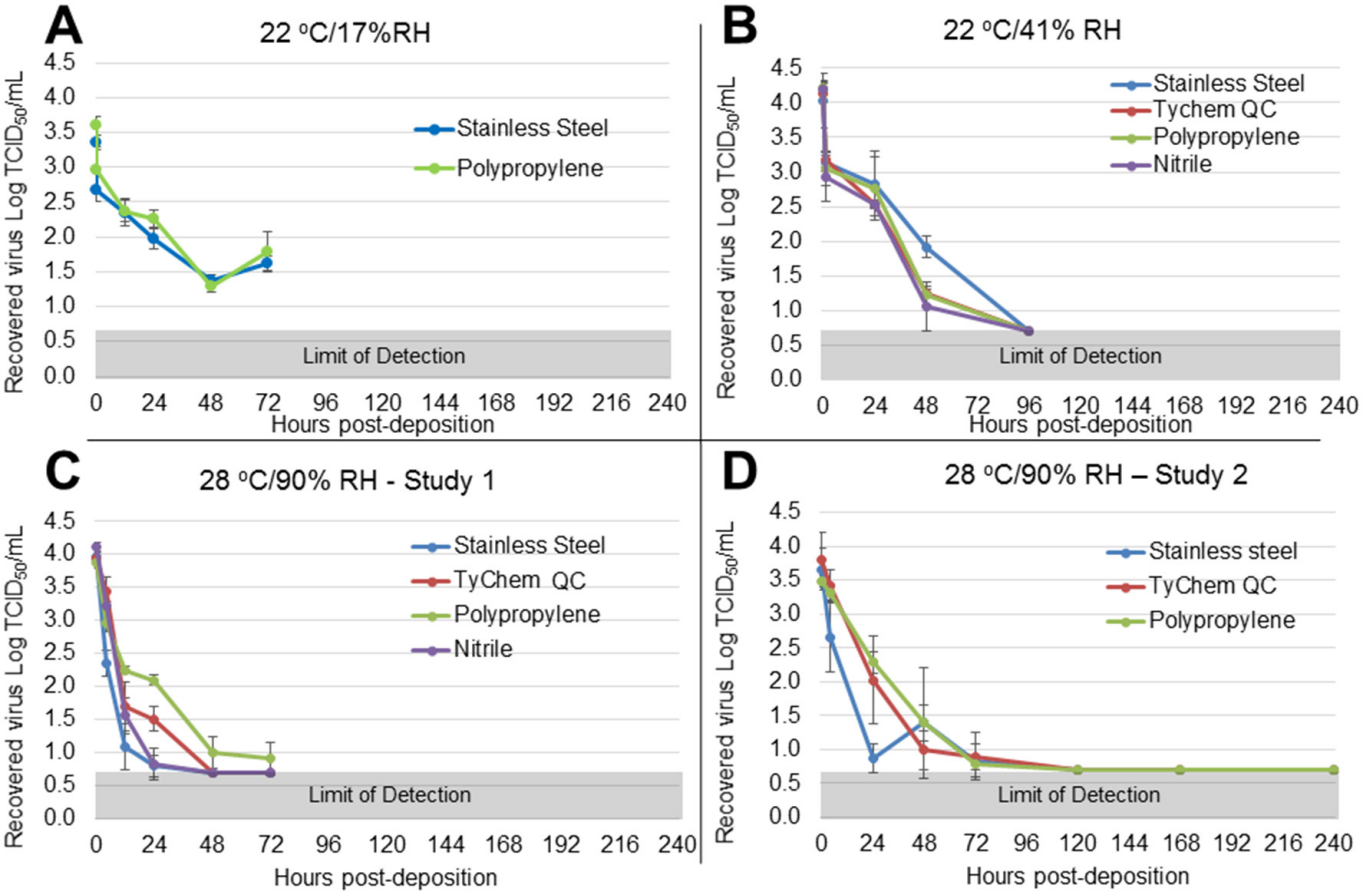
EBOV/Mak-C05 Surface Persistence in Cell Culture Media at Three Different Environments. The surface-specific decrease in EBOV/Mak-C05 viability in cell culture media was measured at (A) 22°C/17% RH (stainless steel and polypropylene only), (B) 22°C/41% RH, or (C-D) 28°C/90% RH. The microtitration assay limit of detection (0.7 Log TCID_50_/mL) is indicated by a gray bar at the bottom of each graph. The apparent increase at the 72 h timepoint in panel A is most likely due to experimental variation in replicate samples and not an increase in viral titer. The graphs in panels C-D are derived from two separate studies, as described in the methods section.

**Fig 4:**
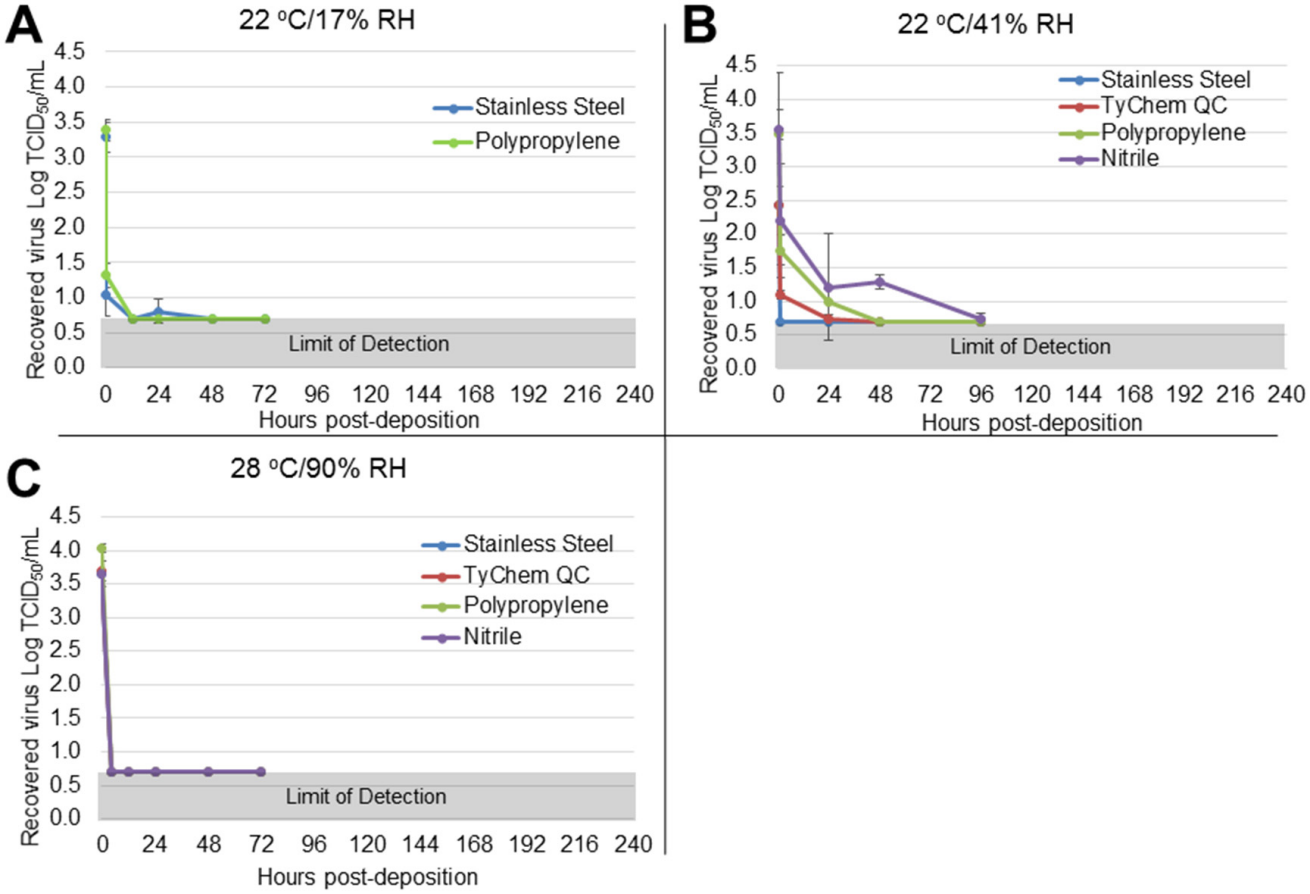
EBOV/Mak-C05 Surface Persistence in Simulated Vomit at Three Different Environments. The surface-specific decrease in EBOV/Mak-C05 viability in simulated vomit was measured at (A) 22°C/17% RH (stainless steel and polypropylene only), (B) 22°C/41% RH, or (C) 28°C/90% RH. The microtitration assay limit of detection (0.7 Log TCID_50_/mL) is indicated by a gray bar at the bottom of each graph.

Of the relevant human fluid matrices tested, EBOV/Mak-C05 persisted longest in dried blood, with short persistence (<24 h) in simulated vomit, and no persistence in feces. We also observed significant EBOV persistence in cell culture media. Figs 2, 3 and 4 show time-dependent EBOV/Mak-C05 persistence data in blood, cell culture media, and simulated vomit, respectively, on stainless steel, TyChem QC, polypropylene, and nitrile, at each test environment. The amount of virus recovered from coupons (Log TCID_50_/mL) is plotted v. timepoint (post-sample deposition), and surface identity is indicated for each graph. The T = 0 timepoints for each graph represent mean (n = 3) pre-drying virus viability values, and the subsequent points show mean (n = 3) post-drying virus viability after aging at the indicated environments. Table 1 summarizes matrix-dependent virus viability decay rates and half-lives, derived from the data shown in Figs 2, 3 and 4 using a least squares data fit, for EBOV/Mak-C05 in human whole blood, cell culture media, and simulated vomit, respectively. In dried blood, EBOV/Mak-C05 persisted longest at 28°C/90% RH (mean half-life = 25 h) and shortest at 22°C/17% RH (mean half-life = 9 h). EBOV/Mak-C05 mean decay rates in cell culture media ranged from 10–28 hours with no observed significant environment-dependency. In simulated vomit, EBOV/Mak-C05 persisted longest at 22°C/41% RH (half-life = 11 h); however, because of the limited number of timepoints containing viable virus, the half-life 95% confidence intervals encompassed a wide range (6–40 h), which reduces the reliability of the mean half-live value. For this matrix, no persistence was observed at 28°C/90% RH. Decay rates were derived using all timepoints from immediately post-drying to 240 h or until the assay limit of detection was reached. Decay rates do not include pre-drying timepoints. Because infectious virus was not recovered from wet or dry feces on any surface at any environment, data for this matrix are not shown and no further studies were performed with feces. We performed an Analysis of Variance (ANOVA) on data from cell culture media and blood persistence studies to identify significant effects of surface, matrix, or environment on EBOV surface persistence. Data acquired from simulated vomit were removed prior to ANOVA analysis, because the rapid viability loss in this matrix made it difficult to estimate reliable decay rates. No analysis was performed on feces, because no viable virus was recovered from this matrix at any timepoint or environment. ANOVA indicated that the matrix type significantly influenced EBOV/Mak-C05 persistence (*P* = 0.002), while non-porous test surface type did not (*P* = 0.09), in agreement with a previous study that demonstrated surface type did not significantly influence persistence in cell culture media [20]. Environmental conditions had a significant effect on EBOV persistence only in dried blood.

**Table 1:** EBOV Viability Decay Rates and Half-lives for Each Test Matrix and Environment. A least-squares method was used to calculate the slope of the line defined by the post-drying surface-averaged points for each matrix and environment. This slope was used to calculate daily decay rate means (reduction in EBOV LogTCID_50_/day) and 95% confidence interval (CI) for EBOV/Mak-C05 and EBOV/Yam-May. Mean half-lives (in hours) and ranges for each matrix and environment are indicated in bold. ND = not determined. NP = no persistence.

	Variant—EBOV/Mak-C05			Variant—EBOV/Yam-May		
	Test Matrix—Media	Test Matrix—Blood	Test Matrix—Simulated Vomit	Test Matrix—Media	Test Matrix—Blood	Test Matrix—Simulated Vomit
**Environment** 22°C/17% RH	0.41 [0.29, 0.53] **18 h (14–25 h)**	0.79 [0.66, 0.91] **9 h (8–11 h)**	0.09 8 h*	ND	ND	ND
**Environment** 22°C/41% RH	0.62 [0.55, 0.70] **12 h (10–13 h)**	0.63 [0.55, 0.71] **11 h (10–13 h)**	0.66 [0.18, 1.2] **11 h (6–40 h)**	0.59 [0.52, 0.66] **12 h (11–14 h)**	0.62 [0.45, 0.80] **12 h (9–16 h)**	NP
**Environment** 28°C/90% RH	0.45 [0.36, 0.55] **16 h (13–20 h)**	0.29 [0.26, 0.32] **25 h (23–28 h)**	NP	0.57 [0.45, 0.69] **13 h (10–16 h)**	0.24 [0.20, 0.28] **30 h (26–36 h)**	NP

*Because of the low persistence of EBOV in simulated vomit, the decay half-life was determined using two post-drying points only, and no confidence interval was calculated.

**Fig 5:**
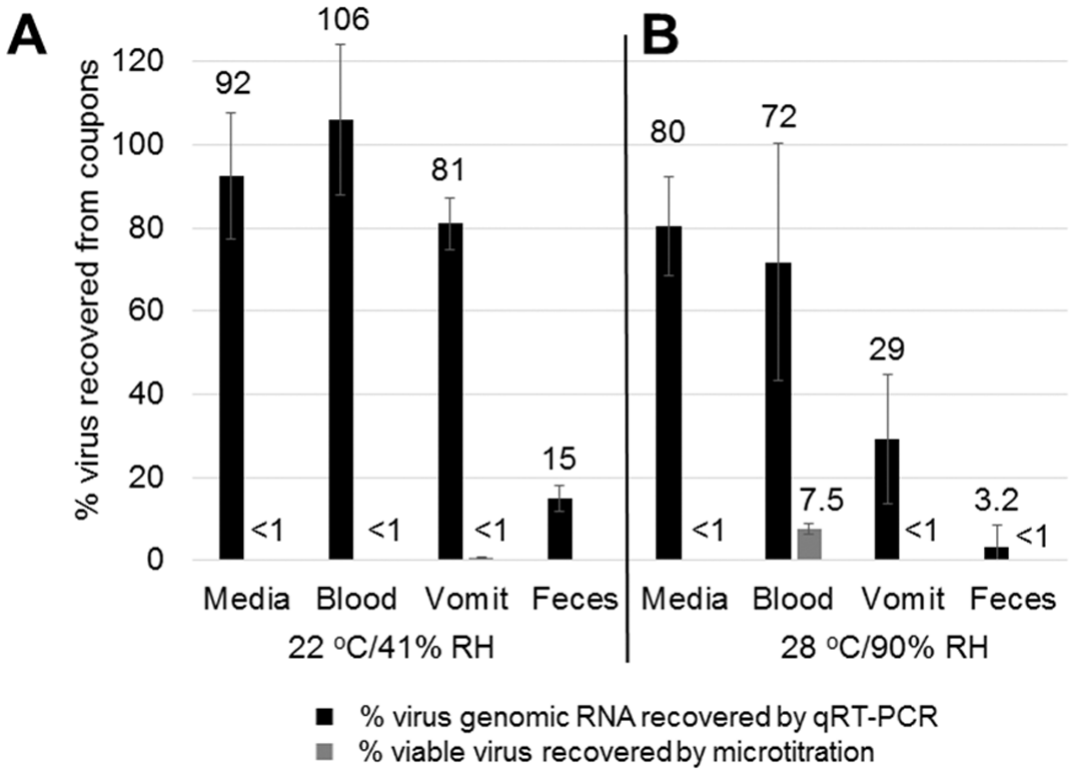
Comparison of EBOV/Mak-C05 Recovery by qRT-PCR and Viability by Microtitration Assay. The virus samples recovered from test surface coupons from Figs 2, 3 and 4 were analyzed by both qRT-PCR and virus microtitration assay to determine whether virus material was efficiently recovered at low viability timepoints (n = 3). qRT-PCR analysis was performed on samples aged at (A) 22°C/41% RH, or (B) 28°C/90% RH. The ratio of virus genomic copies recovered from timepoints at the microtitration limit of detection to recovery from timepoints immediately post-drying was determined and plotted as a percentage (black bars). This was compared to the virus viability ratio from the same timepoints, as determined by microtitration assay (Log TCID_50_/mL) (gray bars). Actual mean percent recovery values are shown above each bar. Because no viable virus was detected at any timepoint in feces and % viable virus could not be calculated, % viable virus recovered in this matrix is indicated at <1%.

Quantitative reverse-transcription PCR was performed on a representative number of recovered samples from studies performed at 22°C/41% RH and 28°C/90% RH to quantitate total virus genomic copies recovered from coupons. The total number of virus genomic copies recovered from coupons was determined from pre-drying timepoints and was compared to the virus genomic copies recovered from samples whose viability was at the limit of microtitration assay detection (for cell culture media, simulated vomit, and feces samples) or up to 96 h post-deposition (for blood, where the limit of detection was not reached). The percentage of recovered virus genomic copies was calculated from the ratio of post-deposition:pre-drying timepoints and plotted next to the percentage of infectious virus recovered from the same samples. Fig 5 shows the comparison of percent recovered virus measured both by qRT-PCR and viability assays, and demonstrates that the amount of virus recovered from test surfaces as measured by qRT-PCR did not significantly differ between the paired high and low viability samples in blood and cell culture media. This indicates that the decay in virus viability measured by the microtitration assay reflects a loss of viability and not a reduction in the amount of virus material recovered. Samples in dried cell culture media and dried blood show >70% of virus genome recovery (compared to wet samples) at both environments shown. However, at 28°C/90% RH, the samples from dried simulated vomit show >3-fold reduction in the number of genome copies recovered from high and low viability timepoints. Similarly, samples from feces demonstrated a >6-fold reduction in total recovered virus genome copies from wet samples and >30-fold reduction in total recovered virus genome copies from dried samples, at both environments shown. These data suggest that simulated vomit, and to a greater extent feces, contribute to a reduction in total virus recovery, possibly through inactivation of virus and rapid degradation of virus genomic RNA, but additional studies must be performed to discern this.

**Fig 6:**
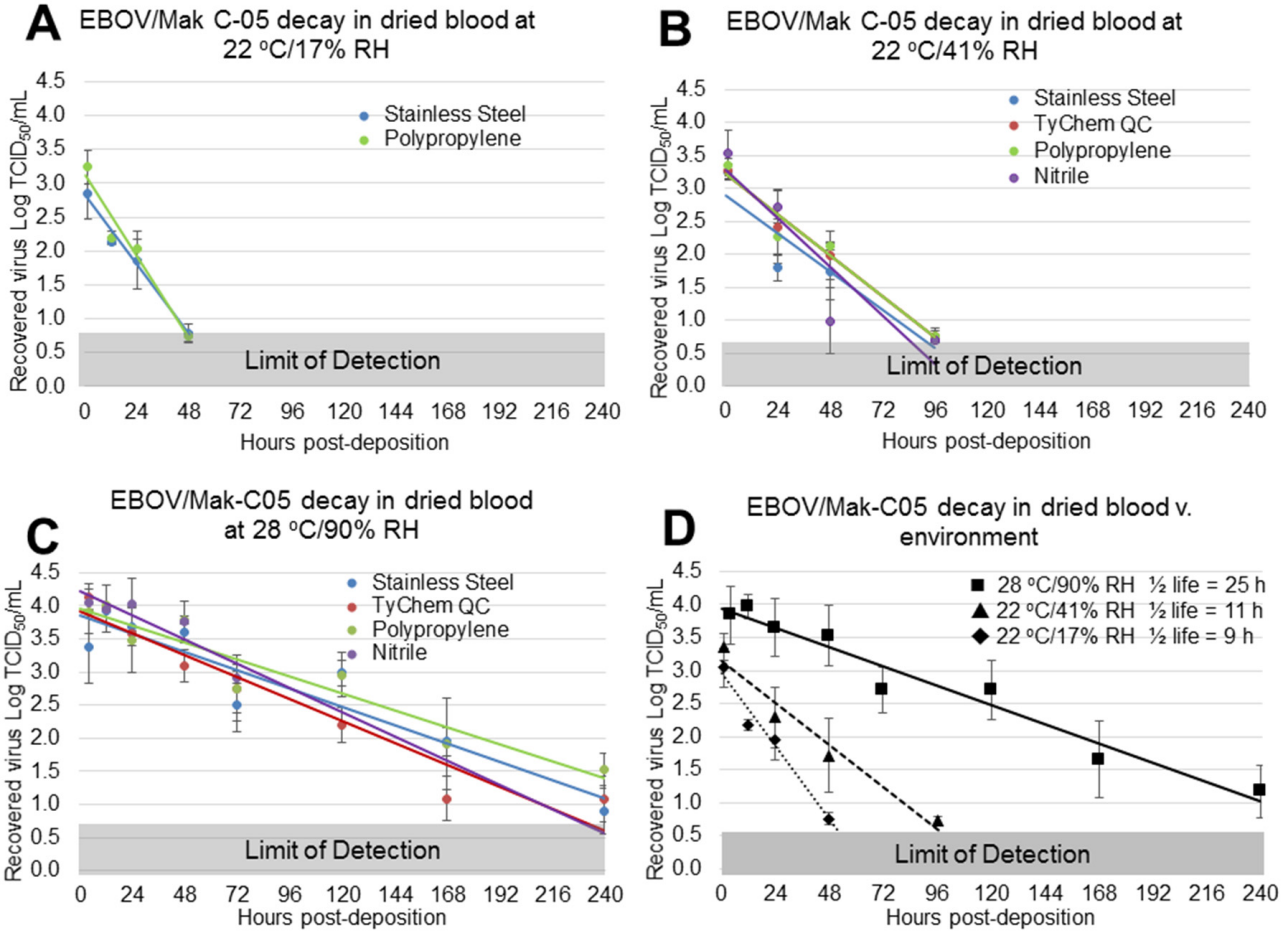
Environment-Dependent EBOV Viability Decay and Half-lives in Dried Human Whole Blood. (A-C) The surface-specific decrease in EBOV/Mak-C05 viability in dried blood was measured at (A) 22°C/17% RH for 72 h, (B) 22°C/41% RH for 96 h, or (C) 28°C/90% RH for 240 h. (D) Because ANOVA indicated that surface type did not significantly affect virus persistence, EBOV/Mak-C05 viability values from all surfaces were combined for each timepoint (a minimum of n = 6 for each timepoint) and the mean surface viability was plotted v. post-deposition time. A least-squares analysis was used to plot surface-independent virus viability decay at each environment (∎ 28°C/90% RH, solid line, ▲ 22°C/41% RH, dashed line, ◆22°C/17% RH, dotted line) and to derive surface-independent decay rates. These rates were used to calculate mean virus viability half-lives and 95% confidence intervals at each environment, and can be found in Table 1. The microtitration assay limit of detection (0.7 Log TCID_50_/mL) is indicated by a gray bar at the bottom of panels A-D.

The environmental condition significantly affected virus persistence only in dried blood (*P* = 0.02). Because dried blood was the only human fluid matrix in which EBOV/Mak-C05 consistently persisted longer than 24 h, the additional studies focused on EBOV persistence in this matrix. Surface-specific viability decay rates in dried blood at the three relevant environments were derived from the persistence study data using a least squares data fit method (Fig 6A–6C), and surface-specific virus viability decay trendlines at each environment were plotted using points from immediately post-dry to 240 h, or until the assay limit of detection was reached. The change in virus viability (Log TCID_50_/mL) is plotted v. post-deposition time, and surface identity is indicated in each panel. Because ANOVA indicated that persistence in dried blood was surface-independent, the surface data at each timepoint were combined to increase the power of the statistical analysis and further refine the decay rates in dried blood. Fig 6D shows that in dried blood, EBOV/Mak-C05 persisted longest at 28°C/90% RH.

### EBOV/Mak-C05 is able to withstand the stresses associated with drying in blood significantly better than EBOV/Yam-May

**Fig 7:**
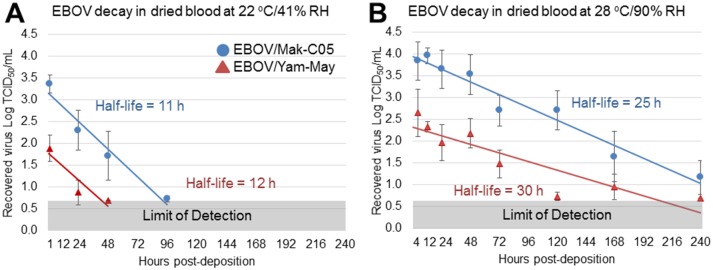
Comparative EBOV Viability Decay Rates and Stability in Dried Human Whole Blood. (A-B) Surface-composite decay plots in dried blood for EBOV/Mak-C05 and EBOV/Yam-May at 22°C/41% RH (A), and 28°C/90% RH (B). Viability values from stainless steel, TyChem QC, polypropylene (for both EBOV variants), and nitrile (for EBOV/Mak-C05 only) surfaces were combined at each timepoint (a minimum of n = 9 for each timepoint) and the mean viability was plotted v. post-drying time, using a least squares analysis, to derive surface-independent decay rates for each virus variant. These rates were used to calculate mean virus viability half-lives (in hours) at the two different environments in dried blood, which are indicated next to each decay line. Pre-drying timepoints are not included. Virus variants are indicated in the legend in panel A (EBOV/Mak-C05, blue dots, EBOV/Yam-May, red triangles).

**Fig 8:**
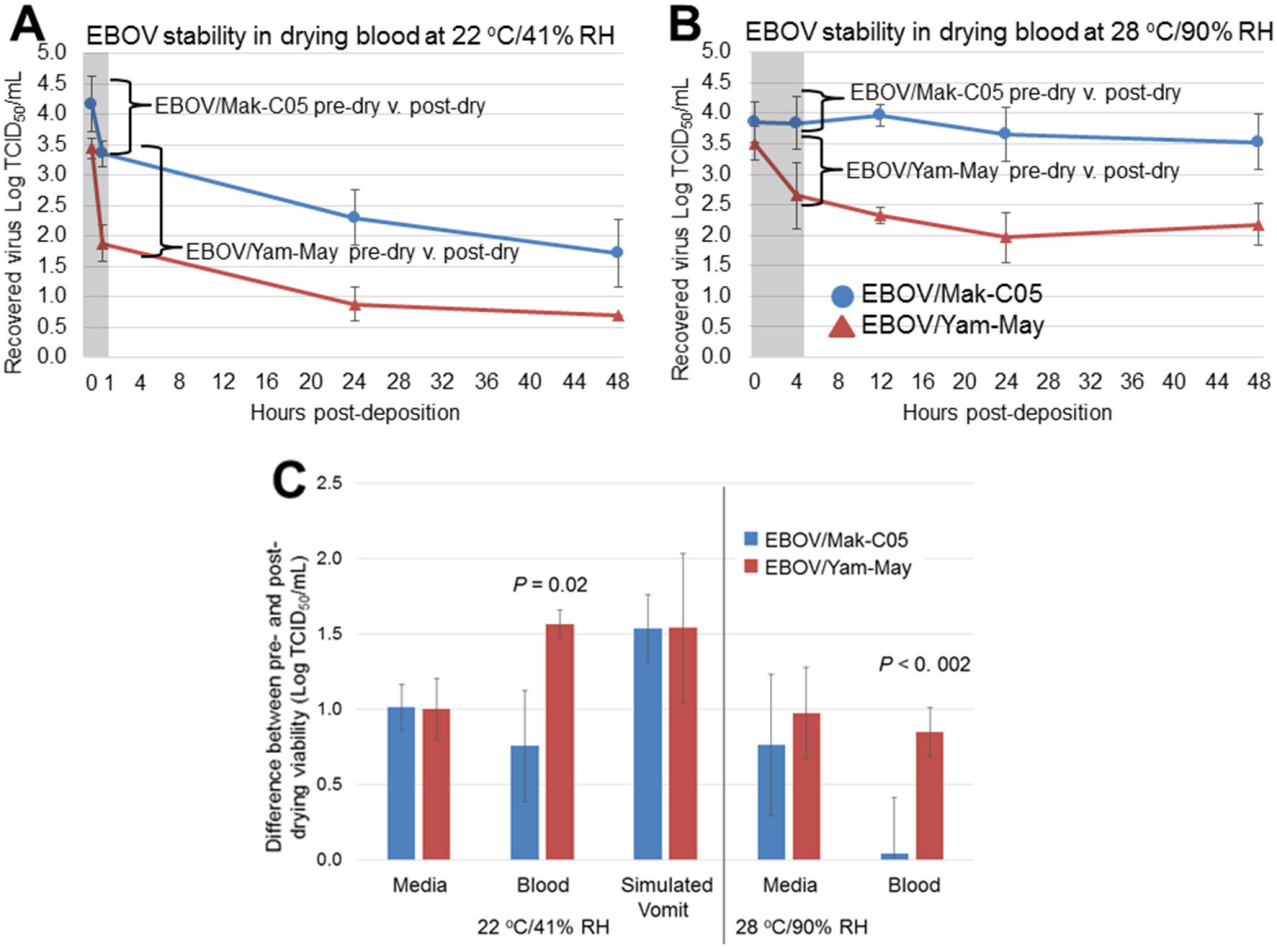
Drying in Blood Reduces Viability of EBOV/Yam-May More Than EBOV/Mak-C05. Drops of human whole blood (10 μL) spiked with either EBOV/Mak-C05 (blue) or EBOV/Yam-May (red) were spotted onto surface coupons. The spots were either recovered immediately (pre-dry) or dried at (A) 22°C/41% RH for one hour, or (B) 28°C/90% RH for four hours, and then recovered (post-dry). Viable virus titers in each recovered sample were determined, and the difference between pre- and post-dry samples were compared. The T = 0 timepoints (n = 3) for each graph represent pre-drying virus viability values, and the second point (one hour post-deposition at 22°C/41% RH and four hours post-deposition at 28°C/90% RH) shows virus viability immediately post-drying (highlighted in gray). Virus variants are indicated in the legend in panel A (EBOV/Mak-C05, blue dots, EBOV/Yam-May, red triangles). (C) The difference in the reduction in viable EBOV during drying in cell culture media, whole blood, and simulated vomit is shown.

To determine whether the difference in magnitude between the current and previous outbreaks might be partially due to a difference in EBOV variant stability, we next investigated whether EBOV/Mak-C05 viability decay rates differed significantly from those of EBOV/Yam-May, which is the prototype virus variant from the first documented EBOV outbreak in Yambuku, DRC [22]. EBOV/Yam-May viability decay rates in dried blood did not significantly differ from the EBOV/Mak-C05 decay rates (Table 1, Fig 7A and 7B). Persistence of EBOV/Yam-May in dried simulated vomit and feces also did not significantly differ from that of EBOV/Mak-C05 in that short persistence was observed in vomit and no persistence was observed in feces (Tables E-G in S1 File). However, during the drying process in blood, we observed a difference in loss of viral titer between EBOV/Mak-C05 and EBOV/Yam-May (Fig 8A and 8B). This difference was significant (*p* = 0.002) and most pronounced at 28°C/90% RH, where 0.04 Log TCID_50_/mL of the deposited EBOV/Mak-C05 was lost during drying, compared to 0.8 Log TCID_50_/mL lost for EBOV/Yam-May (Fig 8C). This indicates that EBOV/Mak-C05 is able to withstand the stresses associated with drying in blood significantly better than EBOV/Yam-May, especially at environments representative of an open-air EBOV treatment center in Western Africa. At 22°C/41% RH, which represents a climate-controlled hospital environment, the difference was less pronounced but still significant (*p* = 0.02), with 0.8 Log TCID_50_/mL of the deposited EBOV/Mak-C05 lost during drying, compared to 1.6 Log TCID_50_/mL lost for EBOV/Yam-May. No significant differences were observed in other matrix/environment combinations (Fig 8C). EBOV viability losses due to drying in simulated vomit at 28°C/90% RH are not graphed because no significant EBOV persistence was observed in post-drying samples at this environment. The results suggest that, with equivalent starting titers, viable EBOV/Mak-C05 will persist longer in dried blood on non-porous surfaces than EBOV/Yam-May.

## Discussion

This study provides empirically-derived EBOV viability decay rates in human clinical fluids, on hospital, airplane, and PPE surfaces, at three relevant environments, and identified differences in the current outbreak and prototypical EBOV virus variant persistence phenotypes that may contribute to increased communicability of EBOV/Mak-C05 when contaminated dried blood is present. Our results show that, while the decay rates of the two virus variants are the same post-drying, EBOV/Mak-C05’s higher persistence during the drying process in blood results in higher post-drying titers in this matrix, compared to EBOV/Yam-May. This may result in a greater potential opportunity for fomite-mediated transmission. These data can be used to inform and improve risk assessments regarding the handling of contaminated fomites and/or remediation of contaminated facilities [23].

Prior filovirus persistence studies have been performed, but they have not directly compared the persistence of virus variants, evaluated persistence in more than one clinical human fluid matrix, or examined relevant surfaces in applicable environmental conditions, making it difficult to draw conclusions from incomparable data. Belanov *et al*. assessed the persistence of Marburg virus spiked into human blood and on contaminated surfaces (cotton wool, stainless steel and glass) at room temperature and 80% RH [24], in human blood only. The authors found that virus viability persisted in dried human blood for up to five days with a starting virus concentration of 10^5^ guinea pig LD_50_/mL. Piercy *et al*. evaluated the surface persistence of EBOV/Yambuku-Ecran (previously designated E718 and an isolate from the 1976 outbreak in DRC) in cell culture media, and demonstrated that viable virus persisted up to 50 days on glass at 4°C with no persistence observed at ambient temperature [19]. Our study demonstrated EBOV persistence up to 96 h in cell culture media at similar ambient environmental conditions tested by Piercy *et al*. Sagripanti *et al*. investigated the persistence of EBOV/Kikwit in cell culture media on glass at 20–25°C and 30–40% RH, and reported that EBOV decayed at a surface-independent rate of 0.68 Log reduction in virus titer per day (half-life = 11 h) [20]. This agrees with the decay rate from our study (0.62 Log/day reduction, half-life = 12 h) in cell culture media at similar environmental conditions. It should be noted that cell culture media is not a clinically-relevant matrix, but instead it served to bridge the results of this study to previously published studies [19–21]. Cook *et al*. demonstrated that EBOV/Mak-C05, when spiked into a simulated organic soil matrix, persisted longer than 192 h on stainless steel and plastic at 21°C/30% RH, with much shorter persistence on porous textiles [25], in agreement with our results in samples containing whole blood. Lastly, a recent study by Fisher *et al*. [21] determined that, in dried cell culture media, EBOV/Mak-C07 remained viable for 14 days at 21°C/40% RH but only for three days at 27°C/80% RH, on Tyvek. They demonstrated that, at 21°C/40% RH in drying, spiked human blood, EBOV/Mak-C07 remained viable for less than seven days, which agrees with our results. They also showed that EBOV viability decay in blood was independent of environment, which disagrees with our findings. However, Fischer *et al*. did not provide a sufficient description of their methods to accurately reproduce their experiments. For example, they did not describe whether they dried their samples at ambient conditions prior to transferring to the storage environment or dried their samples at the relevant environmental condition (as was done in this study). The findings from each of the previous EBOV stability and persistence studies are summarized in Table H in S1 File. Differences between the results from this study and those of previous studies may be due to differing experimental methods, EBOV variants tested, or test parameters. Overall, this study significantly adds to the understanding of EBOV persistence by determining persistence in more than one clinical human fluid (i.e., blood, vomit, and feces), confirming non-porous surface-independent decay, and providing defined decay rates for multiple relevant environments. Most importantly, this is the only study to compare more than one EBOV variant (EBOV/Makona-C05 and EBOV/Yam-May) in a single study and identify significant persistence differences, specifically in human blood.

Our data show that EBOV does not persist in human feces, indicating that feces may be virucidal. Feces also decreased the sensitivity of the microtitration assay, making detection of viable virus difficult. The lack of detectable infectious virus in both the wet and dry feces matrix that we tested indicates that this matrix is particularly detrimental to virus survival. However, it should be noted that the feces used in this study may not be representative of the diarrhea associated with EVD, which has been characterized as extremely watery and “cholera-like” [26], whereas we utilized human feces from non-EBOV infected humans categorized as loose stool; therefore, virus persistence in the feces of EBOV-infected patients may differ from that observed in this study. Although the presence of blood in either vomit or feces of infected patients has been reported [26] and the presence of trace blood may impact the virus viability decay rates in other matrices, matrix mixtures were not evaluated in this study. This is a clear data gap which must be addressed in future studies.

The results of this surface persistence study provide substantial guidance for future EBOV stability and persistence studies. For example, our data do not support the extensive testing of additional inert, non-porous surfaces with blood (because of the lack of surface effect on virus decay), unless specific differences in the new surface properties can be expected to affect the results (e.g., reactive surfaces). Although our initial study design also included a representative airline carpet sample (relevant to international air travel by EVD patients), we were unable to derive consistent data from airline carpet samples because of complications due to both the porous nature of the carpet (which reduced quantitative recovery of EBOV samples) and microtitration assay cytotoxicity from the carpet samples. These negative results suggest that the testing of porous surfaces (i.e., wood and textiles) is likely to present experimental difficulties due to the absorptive surface properties and incomplete virus recovery that may complicate conclusions about virus persistence and decay on these types of surfaces. Thus, additional methods development is required to establish reliable EBOV persistence data for these surfaces. Currently, the Centers for Disease Control and Prevention recommends that non-porous materials that have been contaminated with EBOV should be placed in leak-proof containment and discarded appropriately [27]. By demonstrating highly variable persistence in different human fluid matrices, our results strongly warrant additional EBOV persistence testing in a broader panel of wet and dried human fluid matrices that have also been shown to harbor infectious virus [19–21, 25, 28].

In summary, EBOV survives longest in dried human blood, has little survival in dried simulated vomit, and does not survive in normal feces, making infected blood the human fluid of greatest concern. Importantly, the Ebola virus responsible for the most recent outbreak survives the drying process in blood better than the virus responsible for the original Ebola outbreak in 1976, and therefore may persist longer, depending on the environment. This could increase the chances of exposure to this virus from surfaces contaminated with human blood, depending on the environment, may partially explain the magnitude of the most recent outbreak, and may impact future outbreak management.

## Supporting Information

S1 File Table A: EBOV/Mak-C05 Surface Persistence Study Performed at 22°C/41% RH—Raw Data. Table B: EBOV/Mak-C05 Surface Persistence Study 1 Performed at 28°C/90% RH—Raw Data. Table C: EBOV/Mak-C05 Surface Persistence Study 2 Performed at 28°C/90% RH—Raw Data. Table D: EBOV/Mak-C05 Surface Persistence Study Performed at 22°C/17% RH—Raw Data. Table E: EBOV/Yam-May Surface Persistence Study Performed at 22°C/41% RH—Raw Data. Table F: EBOV/Yam-May Surface Persistence Study 1 Performed at 28°C/90% RH—Raw Data. Table G: EBOV/Yam-May Surface Persistence Study 2 Performed at 28°C/90% RH—Raw Data. In Tables A-G in S1 File, GMEM = cell culture media matrix; Blood = human whole blood matrix; SGFM = simulated gastric fluid with 2% milk (simulated vomit matrix); Feces = pooled human feces matrix from healthy patients; R1-3 = replicates 1–3; Numerical values in each table represent the virus Log TCID_50_/mL for each replicate recovered from surface coupons; Numbers in red indicate values at the microtitration assay limit of detection of 0.7 (for GMEM, Blood, or SGFM) or 1.2 Log TCID_50_/mL (for Feces). Table H. Summary of Prior EBOV Persistence Studies and Comparison to this Study. * indicates that values were calculated from Log titer loss over 14 days; # indicates that, because ANOVA indicated no surface-dependent effects on EBOV decay, values on different surfaces were combined to calculate surface-independent decay rates.
(DOCX)
