# Supplementary material for: Differences in the Comparative Stability of Ebola Virus Makona-C05 and Yambuku-Mayinga in Blood
Source: PLoS One. 2016 Feb 5;11(2):e0148476. doi: 10.1371/journal.pone.0148476 (PMC4744009; doi:10.1371/journal.pone.0148476)
Supplement: S1 File — Table A: EBOV/Mak-C05 Surface Persistence Study Performed at 22°C/41% RH—Raw Data. Table B: EBOV/Mak-C05 Surface Persistence Study 1 Performed at 28°C/90% RH—Raw Data. Table C: EBOV/Mak-C05 Surface Persistence Study 2 Performed at 28°C/90% RH—Raw Data. Table D: EBOV/Mak-C05 Surface Persistence Study Performed at 22°C/17% RH—Raw Data. Table E: EBOV/Yam-May Surface Persistence Study Performed at 22°C/41% RH—Raw Data. Table F: EBOV/Yam-May Surface Persistence Study 1 Performed at 28°C/90% RH—Raw Data. Table G: EBOV/Yam-May Surface Persistence Study 2 Performed at 28°C/90% RH—Raw Data. In Tables A-G in S1 File, GMEM = cell culture media matrix; Blood = human whole blood matrix; SGFM = simulated gastric fluid with 2% milk (simulated vomit matrix); Feces = pooled human feces matrix from healthy patients; R1-3 = replicates 1–3; Numerical values in each table represent the virus Log TCID50/mL for each replicate recovered from surface coupons; Numbers in red indicate values at the microtitration assay limit of detection of 0.7 (for GMEM, Blood, or SGFM) or 1.2 Log TCID50/mL (for Feces). Table H. Summary of Prior EBOV Persistence Studies and Comparison to this Study. * indicates that values were calculated from Log titer loss over 14 days; # indicates that, because ANOVA indicated no surface-dependent effects on EBOV decay, values on different surfaces were combined to calculate surface-independent decay rates. (DOCX) [file pone.0148476.s001.docx]

**Title:** Differences in the comparative stability of Ebola virus Makona-C05 and Yambuku Mayinga in blood

**Authors:** Michael Schuit^1^, David M. Miller^1^, Mary S. Reddick-Elick^1^, Carly B. Wlazlowski^1^, Claire Marie Filone^1^, Artemas Herzog^2^, Leremy Colf^3^, Victoria Wahl-Jensen^1^, Michael Hevey^1^, and James W. Noah^1^*

**Affiliation:** ^1^National Biodefense Analysis and Countermeasures Center, Frederick, MD 21702, USA.

^2^Censeo Consulting Group, Washington DC, 20005, USA.

^3^Department of Homeland Security (DHS) Science and Technology Directorate, Washington DC, USA.

*Corresponding author. Email: james.noah@nbacc.dhs.gov

## **Supporting Information - Raw Data Tables**

**Data Key**

- GMEM = cell culture media matrix
- Blood = human whole blood matrix
- SGFM = simulated gastric fluid with 2% milk (simulated vomit matrix)
- Feces = pooled human feces matrix from healthy patients
- R1-3 = replicates 1-3
- Numerical values in each table represent the virus Log TCID_50_/mL for each replicate recovered from surface coupons.
- Numbers in red indicate values at the microtitration assay limit of detection of 0.7 (for GMEM, Blood, or SGFM) or 1.2 Log TCID_50_/mL (for Feces).

Table A. EBOV/Mak-C05 Surface Persistence Study Performed at 22 °C/41% RH – Raw Data.

Table B. EBOV/Mak-C05 Surface Persistence Study 1 Performed at 28 °C/90% RH – Raw Data.

Table C. EBOV/Mak-C05 Surface Persistence Study 2 Performed at 28 °C/90% RH – Raw Data.

Table D. EBOV/Mak-C05 Surface Persistence Study Performed at 22 °C/17% RH – Raw Data.

Table E. EBOV/Yam-May Surface Persistence Study Performed at 22 °C/41% RH – Raw Data.

Table F. EBOV/Yam-May Surface Persistence Study 1 Performed at 28 °C/90% RH – Raw Data.

**Table G. EBOV/Yam-May Surface Persistence Study 2 Performed at 28 °C/90% RH – Raw Data.**

**Table H. Summary of Prior EBOV Persistence Studies and Comparison to this Study.**

Table A. EBOV/Mak-C05 Surface Persistence Study Performed at 22 °C/41% RH – Raw Data.

|  | | | | | |  |  |  |  | **Time (h)** | |  |  |  |  |  |
| --- | --- | --- | --- | --- | --- | --- | --- | --- | --- | --- | --- | --- | --- | --- | --- | --- |
|  |  | **0** | | | **1** | | | **24** | | | **48** | | | **96** | | |
| **Matrix** | **Surface** | **R1** | **R2** | **R3** | **R1** | **R2** | **R3** | **R1** | **R2** | **R3** | **R1** | **R2** | **R3** | **R1** | **R2** | **R3** |
| **GMEM** | Stainless Steel | 4.3 | 4.2 | 3.6 | 3.0 | 3.2 | 3.2 | 3.1 | 2.3 | 3.1 | 2.1 | 1.8 | 1.8 | 0.7 | 0.7 | 0.7 |
|  | TyChem^TM^ QC | 4.3 | 4.1 | 4.0 | 3.2 | 3.1 | 3.3 | 2.6 | 2.6 | 2.5 | 1.2 | 1.3 | 1.3 | 0.7 | 0.7 | 0.7 |
|  | Polypropylene | 4.1 | 4.3 | 4.3 | 3.3 | 3.0 | 2.8 | 2.8 | 3.2 | 2.3 | 1.3 | 1.3 | 1.1 | 0.7 | 0.7 | 0.7 |
|  | Nitrile | 4.1 | 4.3 | 4.2 | 3.3 | 2.9 | 2.6 | 2.5 | 2.6 | 2.5 | 1.4 | 1.1 | 0.7 | 0.7 | 0.7 | 0.7 |
|  |  |  |  |  |  |  |  |  |  |  |  |  |  |  |  |  |
| **Blood** | Stainless Steel | 4.1 | 3.4 | 3.3 | 3.1 | 3.4 | 3.3 | 2.0 | 1.8 | 1.6 | 2.1 | 1.3 | 1.8 | 0.7 | 0.7 | 0.7 |
|  | TyChem^TM^ QC | 4.1 | 4.3 | 4.5 | 3.2 | 3.4 | 3.2 | 2.0 | 2.2 | 3.1 | 1.6 | 2.1 | 2.3 | 0.7 | 0.9 | 0.7 |
|  | Polypropylene | 4.2 | 4.5 | 4.1 | 3.3 | 3.5 | 3.3 | 2.1 | 2.6 | 2.1 | 2.1 | 2.1 | 2.2 | 0.7 | 0.8 | 0.7 |
|  | Nitrile | 4.9 | 4.5 | 4.2 | 3.4 | 3.9 | 3.3 | 3.0 | 2.6 | 2.6 | 0.7 | 1.6 | 0.7 | 0.7 | 0.7 | 0.7 |
|  |  |  |  |  |  |  |  |  |  |  |  |  |  |  |  |  |
| **SGFM** | Stainless Steel | 2.6 | 2.5 | 2.2 | 0.7 | 0.7 | 0.7 | 0.7 | 0.7 | 0.7 | 0.7 | 0.7 | 0.7 | 0.7 | 0.7 | 0.7 |
|  | TyChem^TM^ QC | 0.7 | 3.2 | 3.4 | 1.0 | 1.2 | 1.1 | 0.7 | 0.7 | 0.8 | 0.7 | 0.7 | 0.7 | 0.7 | 0.7 | 0.7 |
|  | Polypropylene | 3.6 | 3.5 | 3.4 | 2.0 | 1.7 | 1.6 | 0.9 | 0.8 | 1.2 | 0.7 | 0.7 | 0.7 | 0.7 | 0.7 | 0.7 |
|  | Nitrile | 2.6 | 4.1 | 4.0 | 2.4 | 2.9 | 1.3 | 2.1 | 0.8 | 0.7 | 1.3 | 1.2 | 1.4 | 0.7 | 0.7 | 0.8 |
|  |  |  |  |  |  |  |  |  |  |  |  |  |  |  |  |  |
| **Feces** | Stainless Steel | 1.2 | 1.2 | 1.2 | 1.2 | 1.2 | 1.2 | 1.2 | 1.2 | 1.2 | 1.2 | 1.2 | 1.2 | 1.2 | 1.2 | 1.2 |
|  | TyChem^TM^ QC | 1.2 | 1.2 | 1.2 | 1.2 | 1.2 | 1.2 | 1.2 | 1.2 | 1.2 | 1.2 | 1.2 | 1.2 | 1.2 | 1.2 | 1.2 |
|  | Polypropylene | 1.2 | 1.2 | 1.2 | 1.2 | 1.2 | 1.2 | 1.2 | 1.2 | 1.2 | 1.2 | 1.2 | 1.2 | 1.2 | 1.2 | 1.2 |
|  | Nitrile | 1.2 | 1.2 | 1.2 | 1.2 | 1.2 | 1.2 | 1.2 | 1.2 | 1.2 | 1.2 | 1.2 | 1.2 | 1.2 | 1.2 | 1.2 |
|  |  |  |  |  |  |  |  |  |  |  |  |  |  |  |  |  |

Table B. EBOV/Mak-C05 Surface Persistence Study 1 Performed at 28 °C/90% RH – Raw Data.

|  | | | | | | |  |  |  |  | **Time (h)** | |  |  |  |  |  |  |  |
| --- | --- | --- | --- | --- | --- | --- | --- | --- | --- | --- | --- | --- | --- | --- | --- | --- | --- | --- | --- |
|  |  | **0** | | | **4** | | | **12** | | | **24** | | | **48** | | | **72** | | |
| **Matrix** | **Surface** | **R1** | **R2** | **R3** | **R1** | **R2** | **R3** | **R1** | **R2** | **R3** | **R1** | **R2** | **R3** | **R1** | **R2** | **R3** | **R1** | **R2** | **R3** |
| **GMEM** | Stainless Steel | 4.1 | 3.9 | 3.8 | 2.6 | 2.3 | 2.2 | 0.7 | 1.3 | 1.3 | 1.0 | 0.7 | 0.7 | 0.7 | 0.7 | 0.7 | 0.7 | 0.7 | 0.7 |
|  | TyChem^TM^ QC | 4.0 | 4.0 | 3.8 | 3.3 | 3.3 | 3.7 | 1.8 | 2.0 | 1.3 | 1.7 | 1.3 | 1.5 | 0.7 | 0.7 | 0.7 | 0.7 | 0.7 | 0.7 |
|  | Polypropylene | 4.0 | 3.8 | 3.8 | 2.8 | 3.0 | 3.1 | 2.2 | 2.3 | 2.3 | 2.1 | 2.1 | 2.2 | 0.9 | 1.3 | 0.8 | 1.2 | 0.8 | 0.7 |
|  | Nitrile | 4.1 | 4.1 | 4.2 | 3.2 | 3.3 | 3.2 | 1.8 | 1.6 | 1.3 | 0.7 | 1.1 | 0.7 | 0.7 | 0.7 | 0.7 | 0.7 | 0.7 | 0.7 |
|  |  |  |  |  |  |  |  |  |  |  |  |  |  |  |  |  |  |  |  |
| **Blood** | Stainless Steel | 3.8 | 3.6 | 4.3 | 3.9 | 3.3 | 3.4 | 3.8 | 3.8 | 4.1 | 3.7 | 3.8 | 3.9 | 3.2 | 3.3 | 3.2 | 2.8 | 2.2 | 2.2 |
|  | TyChem^TM^ QC | 3.8 | 3.8 | 4.1 | 4.2 | 3.6 | 4.5 | 3.9 | 4.1 | 3.8 | 3.6 | 4.0 | 4.2 | 3.8 | 3.4 | 3.3 | 2.8 | 3.1 | 2.6 |
|  | Polypropylene | 3.4 | 4.2 | 3.3 | 3.7 | 4.1 | 3.8 | 3.9 | 4.1 | 4.0 | 4.0 | 4.1 | 3.7 | 3.6 | 3.7 | 3.8 | 3.0 | 2.1 | 2.8 |
|  | Nitrile | 4.0 | 4.0 | 3.6 | 4.1 | 3.9 | 4.1 | 4.3 | 3.6 | 4.0 | 4.2 | 3.6 | 4.3 | 4.1 | 3.6 | 3.6 | 3.0 | 2.9 | 2.8 |
|  |  |  |  |  |  |  |  |  |  |  |  |  |  |  |  |  |  |  |  |
| **SGFM** | Stainless Steel | 3.6 | 3.4 | 4.0 | 0.7 | 0.7 | 0.7 | 0.7 | 0.7 | 0.7 | 0.7 | 0.7 | 0.7 | 0.7 | 0.7 | 0.7 | 0.7 | 0.7 | 0.7 |
|  | TyChem^TM^ QC | 4.1 | 3.4 | 3.6 | 0.7 | 0.7 | 0.7 | 0.7 | 0.7 | 0.7 | 0.7 | 0.7 | 0.7 | 0.7 | 0.7 | 0.7 | 0.7 | 0.7 | 0.7 |
|  | Polypropylene | 4.0 | 4.0 | 4.1 | 0.7 | 0.7 | 0.7 | 0.7 | 0.7 | 0.7 | 0.7 | 0.7 | 0.7 | 0.7 | 0.7 | 0.7 | 0.7 | 0.7 | 0.7 |
|  | Nitrile | 3.4 | 3.7 | 3.8 | 0.7 | 0.7 | 0.7 | 0.7 | 0.7 | 0.7 | 0.7 | 0.7 | 0.7 | 0.7 | 0.7 | 0.7 | 0.7 | 0.7 | 0.7 |
|  |  |  |  |  |  |  |  |  |  |  |  |  |  |  |  |  |  |  |  |
| **Feces** | Stainless Steel | 1.2 | 1.2 | 1.2 | 1.2 | 1.2 | 1.2 | 1.2 | 1.2 | 1.2 | 1.2 | 1.2 | 1.2 | 1.2 | 1.2 | 1.2 | 1.2 | 1.2 | 1.2 |
|  | TyChem^TM^ QC | 1.2 | 1.2 | 1.2 | 1.2 | 1.2 | 1.2 | 1.2 | 1.2 | 1.2 | 1.2 | 1.2 | 1.2 | 1.2 | 1.2 | 1.2 | 1.2 | 1.2 | 1.2 |
|  | Polypropylene | 1.2 | 1.2 | 1.2 | 1.2 | 1.2 | 1.2 | 1.2 | 1.2 | 1.2 | 1.2 | 1.2 | 1.2 | 1.2 | 1.2 | 1.2 | 1.2 | 1.2 | 1.2 |
|  | Nitrile | 1.2 | 1.2 | 1.2 | 1.2 | 1.2 | 1.2 | 1.2 | 1.2 | 1.2 | 1.2 | 1.2 | 1.2 | 1.2 | 1.2 | 1.2 | 1.2 | 1.2 | 1.2 |
|  |  |  |  |  |  |  |  |  |  |  |  |  |  |  |  |  |  |  |  |

Table C. EBOV/Mak-C05 Surface Persistence Study 2 Performed at 28 °C/90% RH – Raw Data.

|  | | | | | | |  |  |  |  |  |  |  | **Time (h)** | |  |  |  |  |  |  |  |  |  |  |
| --- | --- | --- | --- | --- | --- | --- | --- | --- | --- | --- | --- | --- | --- | --- | --- | --- | --- | --- | --- | --- | --- | --- | --- | --- | --- |
|  |  | **0** | | | **4** | | | **24** | | | **48** | | | **72** | | | **120** | | | **168** | | | **240** | | |
| **Matrix** | **Surface** | **R1** | **R2** | **R3** | **R1** | **R2** | **R3** | **R1** | **R2** | **R3** | **R1** | **R2** | **R3** | **R1** | **R2** | **R3** | **R1** | **R2** | **R3** | **R1** | **R2** | **R3** | **R1** | **R2** | **R3** |
| **GMEM** | Stainless Steel | 3.4 | 4.0 | 3.6 | 3.2 | 2.6 | 2.2 | 1.1 | 0.8 | 0.7 | 2.3 | 0.7 | 1.2 | 0.7 | 0.7 | 1.1 | 0.7 | 0.7 | 0.7 | 0.7 | 0.7 | 0.7 | 0.7 | 0.7 | 0.7 |
|  | TyChem^TM^ QC | 3.4 | 3.8 | 4.2 | 3.3 | 3.3 | 3.7 | 2.2 | 2.6 | 1.3 | 1.0 | 0.7 | 1.3 | 0.7 | 0.7 | 1.3 | 0.7 | 0.7 | 0.7 | 0.7 | 0.7 | 0.7 | 0.7 | 0.7 | 0.7 |
|  | Polypropylene | 3.5 | 3.4 | 3.6 | 3.2 | 3.4 | 3.4 | 2.2 | 2.5 | 2.2 | 1.6 | 1.5 | 1.1 | 0.7 | 0.8 | 0.8 | 0.7 | 0.7 | 0.7 | 0.7 | 0.7 | 0.7 | 0.7 | 0.7 | 0.7 |
| **Blood** | Stainless Steel | 3.8 | 4.0 | 3.4 | 3.6 | 3.5 | 2.6 | 3.6 | 3.8 | 3.3 | 4.0 | 4.1 | 3.8 | 3.0 | 2.2 | 2.6 | 2.9 | 3.2 | 2.8 | 1.8 | 1.8 | 2.2 | 1.3 | 0.7 | 0.7 |
|  | TyChem^TM^ QC | 3.8 | 4.2 | 3.3 | 4.0 | 4.2 | 4.4 | 3.1 | 3.3 | 3.3 | 2.8 | 2.4 | 2.8 | 2.3 | 2.9 | 2.8 | 1.9 | 2.4 | 2.3 | 1.3 | 1.3 | 0.7 | 1.3 | 0.7 | 1.2 |
|  | Polypropylene | 4.1 | 4.2 | 4.1 | 4.1 | 4.2 | 3.6 | 2.6 | 3.6 | 3.0 | 3.8 | 3.8 | 3.9 | 2.3 | 3.1 | 3.2 | 2.6 | 3.1 | 3.2 | 2.3 | 2.3 | 1.1 | 1.3 | 1.5 | 1.8 |

Table D. EBOV/Mak-C05 Surface Persistence Study Performed at 22 °C/17% RH – Raw Data.

|  | | | | | |  |  |  |  |  | **Time (h)** | |  |  |  |  |  |  |  |
| --- | --- | --- | --- | --- | --- | --- | --- | --- | --- | --- | --- | --- | --- | --- | --- | --- | --- | --- | --- |
|  |  | **0** | | | **1** | | | **12** | | | **24** | | | **48** | | | **72** | | |
| **Matrix** | **Surface** | **R1** | **R2** | **R3** | **R1** | **R2** | **R3** | **R1** | **R2** | **R3** | **R1** | **R2** | **R3** | **R1** | **R2** | **R3** | **R1** | **R2** | **R3** |
| **GMEM** | Stainless Steel | 3.5 | 3.2 | 3.4 | 2.8 | 2.8 | 2.4 | 2.6 | 2.4 | 2.1 | 2.0 | 1.8 | 2.1 | 1.5 | 1.3 | 1.4 | 1.7 | 1.7 | 1.5 |
|  | Polypropylene | 3.8 | 3.6 | 3.4 | 3.3 | 2.7 | 2.9 | 2.2 | 2.3 | 2.6 | 2.1 | 2.3 | 2.4 | 1.3 | 1.2 | 1.4 | 2.1 | 1.5 | 1.8 |
| **Blood** | Stainless Steel | 3.7 | 3.4 | 3.7 | 2.6 | 2.7 | 3.3 | 2.1 | 2.1 | 2.2 | 1.8 | 2.3 | 1.5 | 0.7 | 0.9 | 0.7 | 0.7 | 0.7 | 0.7 |
|  | Polypropylene | 3.4 | 3.8 | 4.2 | 3.5 | 3.2 | 3.0 | 2.1 | 2.2 | 2.3 | 2.2 | 1.9 | 2.0 | 0.7 | 0.7 | 0.8 | 0.7 | 0.7 | 0.7 |
| **SGFM** | Stainless Steel | 3.1 | 3.6 | 3.2 | 0.7 | 1.3 | 1.1 | 0.7 | 0.7 | 0.7 | 0.7 | 1.0 | 0.7 | 0.7 | 0.7 | 0.7 | 0.7 | 0.7 | 0.7 |
|  | Polypropylene | 3.2 | 3.4 | 3.6 | 1.5 | 1.4 | 1.1 | 0.7 | 0.7 | 0.7 | 0.7 | 0.7 | 0.7 | 0.7 | 0.7 | 0.7 | 0.7 | 0.7 | 0.7 |
| **Feces** | Stainless Steel | 1.2 | 1.2 | 1.2 | 1.2 | 1.2 | 1.2 | 1.2 | 1.2 | 1.2 | 1.2 | 1.2 | 1.2 | 1.2 | 1.2 | 1.2 | 1.2 | 1.2 | 1.2 |
|  | Polypropylene | 1.2 | 1.2 | 1.2 | 1.2 | 1.2 | 1.2 | 1.2 | 1.2 | 1.2 | 1.2 | 1.2 | 1.2 | 1.2 | 1.2 | 1.2 | 1.2 | 1.2 | 1.2 |

Table E. EBOV/Yam-May Surface Persistence Study Performed at 22 °C/41% RH – Raw Data.

|  | | | | | | |  |  |  | **Time (h)** | |  |  |  |  |  |
| --- | --- | --- | --- | --- | --- | --- | --- | --- | --- | --- | --- | --- | --- | --- | --- | --- |
|  |  | **0** | | | **1** | | | **24** | | | **48** | | | **72** | | |
| **Matrix** | **Surface** | **R1** | **R2** | **R3** | **R1** | **R2** | **R3** | **R1** | **R2** | **R3** | **R1** | **R2** | **R3** | **R1** | **R2** | **R3** |
| **Blood** | Stainless Steel | 3.4 | 3.2 | 3.3 | 2.0 | 1.6 | 1.7 | 0.7 | 0.7 | 0.7 | 0.7 | 0.7 | 0.7 | 0.7 | 0.7 | 0.7 |
|  | TyChem^TM^ QC | 3.3 | 3.6 | 3.7 | 2.0 | 2.1 | 1.9 | 0.8 | 1.0 | 1.6 | 0.7 | 0.7 | 0.7 | 0.7 | 0.7 | 0.7 |
|  | Polypropylene | 3.6 | 3.6 | 3.4 | 2.2 | 2.1 | 1.3 | 0.7 | 1.0 | 0.7 | 0.7 | 0.7 | 0.7 | 0.7 | 0.7 | 0.7 |
| **Feces** | Stainless Steel | 1.2 | 1.2 | 1.2 | 1.2 | 1.2 | 1.2 | 1.2 | 1.2 | 1.2 | 1.2 | 1.2 | 1.2 | 1.2 | 1.2 | 1.2 |
|  | TyChem^TM^ QC | 1.2 | 1.2 | 1.2 | 1.2 | 1.2 | 1.2 | 1.2 | 1.2 | 1.2 | 1.2 | 1.2 | 1.2 | 1.2 | 1.2 | 1.2 |
|  | Polypropylene | 1.2 | 1.2 | 1.2 | 1.2 | 1.2 | 1.2 | 1.2 | 1.2 | 1.2 | 1.2 | 1.2 | 1.2 | 1.2 | 1.2 | 1.2 |
| **GMEM** | Stainless Steel | 3.2 | 3.6 | 3.3 | 2.4 | 2.2 | 2.3 | 1.5 | 1.9 | 2.1 | 1.1 | 0.9 | 1.1 | 0.7 | 0.7 | 0.7 |
|  | TyChem^TM^ QC | 3.3 | 3.9 | 3.3 | 2.5 | 3.0 | 2.7 | 1.9 | 1.3 | 1.9 | 1.0 | 0.9 | 0.9 | 0.7 | 0.7 | 0.7 |
|  | Polypropylene | 3.2 | 4.1 | 3.3 | 2.4 | 2.3 | 2.4 | 1.4 | 1.3 | 1.6 | 0.8 | 1.1 | 1.2 | 0.7 | 0.7 | 0.7 |
| **SGFM** | Stainless Steel | 1.2 | 1.2 | 2.6 | 0.7 | 0.7 | 0.7 | 0.7 | 0.7 | 0.7 | 0.7 | 0.7 | 0.7 | 0.7 | 0.7 | 0.7 |
|  | TyChem^TM^ QC | 2.8 | 2.4 | 2.4 | 0.7 | 0.7 | 0.7 | 0.7 | 0.7 | 0.7 | 0.7 | 0.7 | 0.7 | 0.7 | 0.7 | 0.7 |
|  | Polypropylene | 3.1 | 2.3 | 2.2 | 0.7 | 0.7 | 0.7 | 0.7 | 0.7 | 0.7 | 0.7 | 0.7 | 0.7 | 0.7 | 0.7 | 0.7 |

Table F. EBOV/Yam-May Surface Persistence Study 1 Performed at 28 °C/90% RH – Raw Data.

|  | | | | | | |  |  |  |  | **Time (h)** | |  |  |  |  |  |  |  |
| --- | --- | --- | --- | --- | --- | --- | --- | --- | --- | --- | --- | --- | --- | --- | --- | --- | --- | --- | --- |
|  |  | **0** | | | **4** | | | **12** | | | **24** | | | **48** | | | **72** | | |
| **Matrix** | **Surface** | **R1** | **R2** | **R3** | **R1** | **R2** | **R3** | **R1** | **R2** | **R3** | **R1** | **R2** | **R3** | **R1** | **R2** | **R3** | **R1** | **R2** | **R3** |
| **Blood** | Stainless Steel | 3.3 | 3.3 | 3.1 | 1.7 | 2.3 | 2.3 | 2.2 | 2.3 | 2.2 | 1.3 | 1.8 | 1.2 | 2.1 | 2.0 | 2.1 | 1.2 | 1.2 | 1.2 |
|  | TyChem^TM^ QC | 3.3 | 3.6 | 3.2 | 2.6 | 2.4 | 2.3 | 2.4 | 2.2 | 2.4 | 1.5 | 1.3 | 2.0 | 1.8 | 1.9 | 1.7 | 1.7 | 1.8 | 1.9 |
|  | Polypropylene | 3.4 | 3.5 | 3.1 | 2.6 | 2.3 | 2.8 | 2.4 | 2.3 | 2.6 | 2.0 | 2.1 | 1.8 | 1.6 | 1.9 | 2.1 | 1.3 | 1.4 | 1.9 |
| **Feces** | Stainless Steel | 1.2 | 1.2 | 1.2 | 1.2 | 1.2 | 1.2 | 1.2 | 1.2 | 1.2 | 1.2 | 1.2 | 1.2 | 1.2 | 1.2 | 1.2 | 1.2 | 1.2 | 1.2 |
|  | TyChem^TM^ QC | 1.2 | 1.2 | 1.2 | 1.2 | 1.2 | 1.2 | 1.2 | 1.2 | 1.2 | 1.2 | 1.2 | 1.2 | 1.2 | 1.2 | 1.2 | 1.2 | 1.2 | 1.2 |
|  | Polypropylene | 1.2 | 1.2 | 1.2 | 1.2 | 1.2 | 1.2 | 1.2 | 1.2 | 1.2 | 1.2 | 1.2 | 1.2 | 1.2 | 1.2 | 1.2 | 1.2 | 1.2 | 1.2 |
| **GMEM** | Stainless Steel | 3.3 | 3.4 | 3.4 | 2.2 | 2.4 | 1.7 | 0.8 | 1.2 | 1.2 | 0.7 | 0.7 | 0.7 | 0.7 | 0.7 | 0.7 | 0.7 | 0.7 | 0.7 |
|  | TyChem^TM^ QC | 4.0 | 3.5 | 3.6 | 2.2 | 2.2 | 2.6 | 1.6 | 1.4 | 2.1 | 0.8 | 0.7 | 0.9 | 1.7 | 1.2 | 0.9 | 0.7 | 0.7 | 0.7 |
|  | Polypropylene | 3.4 | 3.9 | 3.5 | 2.3 | 2.3 | 2.3 | 1.9 | 1.3 | 2.0 | 1.3 | 1.2 | 1.3 | 1.5 | 0.8 | 0.7 | 0.7 | 0.7 | 0.7 |
| **SGFM** | Stainless Steel | 2.2 | 2.4 | 2.3 | 0.7 | 0.7 | 0.7 | 0.7 | 0.7 | 0.7 | 0.7 | 0.7 | 0.7 | 0.7 | 0.7 | 0.7 | 0.7 | 0.7 | 0.7 |
|  | TyChem^TM^ QC | 2.8 | 2.2 | 2.7 | 0.7 | 0.7 | 0.7 | 0.7 | 0.7 | 0.7 | 0.7 | 0.7 | 0.7 | 0.7 | 0.7 | 0.7 | 0.7 | 0.7 | 0.7 |
|  | Polypropylene | 2.7 | 2.6 | 2.8 | 0.7 | 0.7 | 0.7 | 0.7 | 0.7 | 0.7 | 0.7 | 0.7 | 0.7 | 0.7 | 0.7 | 0.7 | 0.7 | 0.7 | 0.7 |

Table G. EBOV/Yam-May Surface Persistence Study 2 Performed at 28 °C/90% RH – Raw Data.

|  | | | | | | |  |  |  |  |  | **Time (h)** | |  |  |  |  |  |  |  |  |  |  |  |  |
| --- | --- | --- | --- | --- | --- | --- | --- | --- | --- | --- | --- | --- | --- | --- | --- | --- | --- | --- | --- | --- | --- | --- | --- | --- | --- |
|  |  | **0** | | | **4** | | | **24** | | | **48** | | | **72** | | | **120** |  |  | **168** | | | **240** | | |
| **Matrix** | **Surface** | **R1** | **R2** | **R3** | **R1** | **R2** | **R3** | **R1** | **R2** | **R3** | **R1** | **R2** | **R3** | **R1** | **R2** | **R3** | **R1** | **R2** | **R3** | **R1** | **R2** | **R3** | **R1** | **R2** | **R3** |
| **GMEM** | Stainless Steel | 3.4 | 3.9 | 3.8 | 2.3 | 2.2 | 2.8 | 1.3 | 0.7 | 1.1 | 0.7 | 0.7 | 0.7 | 0.7 | 0.7 | 0.7 | 0.7 | 0.7 | 0.7 | 0.7 | 0.7 | 0.7 | 0.7 | 0.7 | 0.7 |
|  | TyChem^TM^ QC | 3.3 | 3.6 | 3.6 | 3.4 | 3.8 | 3.4 | 1.2 | 0.7 | 0.9 | 0.7 | 0.8 | 0.7 | 0.7 | 0.7 | 0.7 | 0.7 | 0.7 | 0.7 | 0.7 | 0.7 | 0.7 | 0.7 | 0.7 | 0.7 |
|  | Polypropylene | 3.4 | 3.6 | 3.3 | 2.4 | 3.0 | 2.8 | 1.4 | 1.4 | 1.3 | 0.9 | 0.8 | 0.7 | 0.7 | 0.7 | 0.7 | 0.7 | 0.7 | 0.7 | 0.7 | 0.7 | 0.7 | 0.7 | 0.7 | 0.7 |
| **Blood** | Stainless Steel | 3.7 | 3.4 | 3.9 | 3.3 | 4.0 | 2.4 | 2.3 | 2.2 | 2.6 | 2.2 | 2.3 | 2.2 | 1.1 | 1.2 | 1.5 | 0.7 | 0.7 | 0.7 | 1.3 | 1.3 | 0.7 | 0.7 | 0.7 | 0.7 |
|  | TyChem^TM^ QC | 3.8 | 3.7 | 3.4 | 2.2 | 2.2 | 3.1 | 2.4 | 2.3 | 1.8 | 2.2 | 2.6 | 2.5 | 1.6 | 1.3 | 1.2 | 0.7 | 0.7 | 0.7 | 0.7 | 0.7 | 0.7 | 0.7 | 0.7 | 0.7 |
|  | Polypropylene | 3.6 | 3.8 | 4.0 | 3.2 | 2.9 | 3.1 | 2.2 | 2.3 | 2.3 | 3.0 | 2.5 | 2.4 | 1.5 | 2.2 | 1.6 | 1.0 | 0.7 | 0.7 | 1.2 | 1.2 | 0.7 | 0.7 | 0.7 | 0.7 |

**Table H. Summary of Prior EBOV Persistence Studies and Comparison to this Study.**

| Reference | Virus | Matrix | Surface | Environment | Decay (Log titer/day) |
| --- | --- | --- | --- | --- | --- |
| Fisher *et al.* | EBOV/Mak-WPGC07 | Cell culture media | Stainless steel | 27 °C/80% RH | 2.22 |
|  |  |  |  | 21 °C/40% RH | 0.77 |
|  |  |  | Plastic | 27 °C/80% RH | 2.22 |
|  |  |  |  | 21 °C/40% RH | 0.55 |
|  |  |  | Tyvek | 27 °C/80% RH | 1.59 |
|  |  |  |  | 21 °C/40% RH | 0.45 |
|  |  | Drying human blood | Plastic | 27 °C/80% RH | 0.67 |
|  |  |  |  | 21 °C/40% RH | 0.67 |
| Piercy *et al.* | EBOV/ Yambuku-Ecran | Cell culture media | Stainless steel | 4 °C | No recovery |
|  |  |  | PVC |  | ~0.29* |
|  |  |  | Glass |  | ~0.07* |
|  |  | Guinea pig sera | Glass |  | ~0.29* |
| Sagripanti *et al.* | EBOV/Kikwit | Cell culture media | Glass | 20-25 °C/30-40% RH | 0.68 |
| Cook *et al.* | EBOV/Mak-C05 | Simulated organic soil load | Stainless steel | 21 °C/30% RH | 0.22 |
|  |  |  | Surgical mask |  | 0.27 |
|  |  |  | Cotton gown |  | 15.35 |
|  |  |  | Plastic gown |  | 0.28 |
| Schuit *et al.* | EBOV/Mak-C05 | Cell culture media | Multiple non-porous surfaces^#^ | 22 °C/17% RH | 0.41 |
|  |  |  |  | 22 °C/41% RH | 0.62 |
|  |  |  |  | 28 °C/90% RH | 0.45 |
|  |  | Dried human blood |  | 22 °C/17% RH | 0.79 |
|  |  |  |  | 22 °C/41% RH | 0.63 |
|  |  |  |  | 28 °C/90% RH | 0.29 |
|  | EBOV/Yam-May | Cell culture media |  | 22 °C/41% RH | 0.59 |
|  |  |  |  | 28 °C/90% RH | 0.57 |
|  |  | Dried human blood |  | 22 °C/41% RH | 0.62 |
|  |  |  |  | 28 °C/90% RH | 0.24 |

* Calculated from Log titer loss over 14 days

# Because ANOVA indicated no surface-dependent effects on EBOV decay, values on different surfaces were combined to calculate surface-independent decay rates
